# Control of Membrane Fouling in Organics Filtration Using Ce-Doped Zirconia and Visible Light

**DOI:** 10.3390/nano9040534

**Published:** 2019-04-03

**Authors:** Fabrício Eduardo Bortot Coelho, Chiara Gionco, Maria Cristina Paganini, Paola Calza, Giuliana Magnacca

**Affiliations:** 1 Department of Chemistry, University of Torino, Via P.Giuria 7, 10125 Torino, Italy; feabfc@gmail.com (F.E.B.C.); chiara.gionco@unito.it (C.G.); mariacristina.paganini@unito.it (M.C.P.); paola.calza@unito.it (P.C.); 2 NIS (Nanostructured Interfaces and surfaces), University of Torino, Via P.Giuria 7, 10125 Torino, Italy

**Keywords:** fouling, humic acid, zirconium dioxide, photocatalysts, cerium, visible light, LED, membrane filtration

## Abstract

Membrane fouling has been a major issue in the development of more efficient water treatment processes. Specifically in surface waters filtration, organic matter, such as humic-like substances, can cause irreversible fouling. Therefore, this study evaluates the activity of a photocatalytic layer composed of Ce-doped zirconia nanoparticles in improving the fouling resistance during filtration of an aqueous solution of humic acid (HA). These nanoparticles were prepared by hydrothermal and sol–gel processes and then characterized. Before the filtration experiments, the photodegradation of HA catalyzed by Ce-doped zirconia nanoparticles in dispersion was studied. It was observed that the sol–gel prepared Ce-ZrO_2_ exhibited higher HA removal in practically neutral pH, achieving 93% efficiency in 180 min of adsorption in the dark followed by 180 min under visible-light irradiation using light-emitting diodes (LEDs). Changes in spectral properties and in total organic carbon confirmed HA degradation and contributed to the proposal of a mechanism for HA photodegradation. Finally, in HA filtration tests, Ce-ZrO_2_ photocatalytic membranes were able to recover the flux in a fouled membrane using visible-light by degrading HA. The present findings point to the further development of anti-fouling membranes, in which solar light can be used to degrade fouling compounds and possibly contaminants of emerging concern, which will have important environmental implications.

## 1. Introduction

As water scarcity becomes more severe and the emission of contaminants of emerging concern (CEC) increases, it is critical to develop more effective and less energy-consuming treatments for drinking and wastewaters [1,2]. In this way, membrane filtration attracts great attention since it has high retention of contaminants [3], and it has already become the established technique for several processes, such as drinking water production from seawater desalination [4]. However, in the operation of membrane systems, fouling is still a major issue, since it decreases the water flux and lifespan of the membranes considerably [2,5,6,7,8]. As a consequence, costs and energy consumption increase, which limits large scale industrial applications of membranes [2,9,10]. There are several processes that cause fouling, such as biological growth, scaling (precipitation of inorganic salts), solid particles, and organic substances (e.g., oils, natural organic matter) [5]. Among the possible sources, the irreversible fouling caused by the adsorption of organic dissolved matter (e.g., humic-like substances) into the membrane surface and pores, is pointed to as a critical issue [11,12,13,14]. Indeed, it usually requires chemical cleaning to recover the original water flux [15]. Therefore, to avoid pre-treatments of the feed stream (e.g., ozonation, flocculation) or membrane cleaning processes (e.g., back pulsing, air scrubbing), a more sustainable approach to controlling fouling would be to functionalize membranes with polymers or nanomaterials, such as carbon nanotubes, zeolite, and iron oxide. This functionalization could act using two different mechanisms. Traditionally, this functionalization has been applied to modify the charge and hydrophobicity of the membrane surface, preventing the adsorption/adhesion of fouling substances [10,16,17,18,19,20]. In a more recent approach, membrane functionalization was added to a photocatalytic layer, which is responsible for the degradation of organic compounds and, therefore, prevents the fouling [21]. Mendret et al. [1] coated a flat-sheet α-Al_2_O_3_ membrane with TiO_2_ and obtained a composite membrane capable of degrading Acid Orange 7 under UV irradiation, preventing flux decrease under filtration.

However, despite TiO_2_’s unique photocatalytic activity and stability [22], this material requires photons in the UV frequencies to create the electron-hole photo-induced separation (i.e., excitation from the valence band (VB) to the conduction band (CB), separated by about 3.2 eV). Hence, this fact practically excludes the use of sunlight as an energy source for TiO_2_. To overcome this limitation, several modifications of TiO_2_ have been studied, such as its doping with transition-metal ions [23], non-metal atoms [24], or supported metal nanoparticles having plasmonic effects [25]. In addition, coupling two different semiconductors also resulted in success [26]. All these systems constitute the second-generation photoactive materials based on titanium dioxide. On the other hand, semiconductors with higher band gap values imply higher electrochemical potentials of excited electrons and holes, which can lead to more effective photocatalytic activity [27]. Therefore, the continuous pursuit of active photocatalysts that work under visible light has addressed the research to develop the third generation of materials not based on TiO_2_ [4,28]. Zirconium dioxide has been rarely applied in photocatalysis because of its wide band gap (ca. 5.0 eV); although some reports are available concerning photo-oxidation of chemicals [29,30,31] and water photosplitting [32]. On the other hand, the band energy levels of ZrO_2_ are suitable for photocatalytic applications because the lowest potential of the CB is −1.0 V (vs. normal hydrogen electrode (NHE), pH 0), much more negative than that of TiO_2_ anatase (−0.1 V), whereas, the highest potential of the VB is +4.0 V, more positive than that of TiO_2_ (+3.1 V). Therefore, ZrO_2_ energetic photo-induced electrons and holes are able to generate reactive oxygen species (ROS), with a great potential to degrade organic compounds or to oxidize the organic molecules directly [33]. In recent works from our group [33,34], doping ZrO_2_ with cerium has introduced intra band gap states between zirconia VB and CB, allowing the new catalyst to absorb visible light through a double jump mechanism. These findings were relevant since they indicated that solar light could be used as a light source for the Ce-ZrO_2_ photocatalytic degradation of organic compounds. Such a process could then be applied in the control of fouling by degrading organic fouling molecules, such as humic-like substances, but also degrading contaminants of emerging concern, which would have important environmental implications.

In the present work, Ce-doped ZrO_2_ was synthesized by hydrothermal and sol–gel processes. The nanopowders obtained were then characterized in terms of crystal structure, morphology, surface area, porosity, surface charge, light absorption, photo-induced charge separation, and ROS generation. Next, since there are no data on the literature about humic acid (HA) photodegradation with Ce-doped zirconia, batch experiments were performed to evaluate the photocatalytic activity of the Ce-ZrO_2_ nanoparticles dispersed in humic acid aqueous solutions under visible light irradiation. Adsorption isotherms were determined and the effects of several parameters (e.g., HA concentration, catalyst dosage, and pH) on HA removal where investigated. The Langmuir–Hinshelwood model was used for kinetic modeling. The changes in spectral properties and in total organic carbon (TOC) were analyzed, confirming that HA is actually degraded during the photocatalytic process. A mechanism for HA photodegradation is proposed. Finally, Ce-doped zirconia nanoparticles were supported in filter papers to produce prototypes of a photocatalytic membrane with improved anti-fouling resistance.

## 2. Materials and Methods

### 2.1. Synthesis and Characterization of Photocatalysts

In the present work, hydrothermal and sol–gel processes were evaluated to prepare zirconia doped with 0.5% molar of cerium. In the hydrothermal synthesis, an aqueous solution of 1.0 M of ZrOCl_2_·8H_2_O (CAS 13520-92-8, purity >98%) and 0.005 M Ce(SO_4_)_2_ (CAS 13454-94-9, essay >99.9%) were mixed and kept under stirring at room temperature. Then, the pH was adjusted to 11 by using an aqueous solution of 4.0 M of NaOH. The resulting solution and the precipitates were then transferred into a 125 mL Teflon-lined stainless steel autoclave, 70% filled, which was heated at 175 °C for 15 h in an oven. The precipitates were, hence, centrifuged and washed three times with deionized water, then dried at 70 °C. This sample was labelled as Ce-ZrO_2_-HYD. For the sol–gel synthesis, 5 mL of zirconium propoxide Zr(OC_3_H_7_)_4_ (CAS 23519-77-9, 70% wt.) were mixed with 5 mL of 2-propanol. Then, 28 mg of Ce(NH_4_)_2_(NO_3_)_6_ (CAS 16774-21-3, purity >98.5%), dissolved in 5 mL of double distilled water, was added to the first solution to start hydrolysis. The resulting gel was kept overnight at room temperature and then dried at 80 °C. After aging at room temperature for 10 days, the xerogel was calcined in a muffle furnace at 500 °C in air for 4 h. This sample was labelled Ce-ZrO_2_-SG. For comparison, pure zirconia samples were prepared by the same hydrothermal and sol–gel procedures, without the addition of cerium, and named ZrO_2_-HYD and ZrO_2_-SG, respectively. All aqueous solutions were prepared using ultrapure water Milli-Q^TM^ (Millipore, Burlington, MA, USA). All chemicals were bought from Sigma-Aldrich (St. Louis, MO, USA) and used without further purification.

Powder X-rays diffraction (XRD) patterns were obtained with the diffractometer PW3040/60 X’Pert PRO MPD (PANalytical, Almelo, The Netherlands), operating at 45 kV, 40 mA, with a Cu Kα radiation source (λ = 1.5418 Å) and a Bragg Brentano geometry over the range 10° < 2θ < 80°.

Nitrogen adsorption–desorption isotherms were obtained (Micromeritics ASAP 2020) for the determination of surface area, using the Brunauer–Emmett–Teller (BET) model, and pore size distribution, using the Barrett-Joyner-Halenda ( BJH) model applied on the desorption branch of the isotherms. Before the adsorption run, all the samples were outgassed at 150 °C for 8 h.

Diffuse reflectance spectroscopy (DRS) data were recorded in the 200 to 800 nm range using the spectrometer Cary 5000 (Varian, Palo Alto, CA, USA), coupled with an integration sphere for diffuse reflectance studies, using Carywin-UV/scan software. A sample of Polytetrafluoroethylene (PTFE) with 100% reflectance was used as the reference. The optical band gap energy has been calculated from the Tauc plot.

Zeta potential measurements were performed on a Zetasizer Nano ZS (Malvern Instrument, Malvern, UK) using principles of laser Doppler velocitometry and phase analysis light scattering (M3-PALS technique). Zero point one percent weight/volume suspensions of nanoparticles were prepared with a NaCl 0.01 M aqueous solution and ultrasonicated for 10 min before the analysis.

### 2.2. Photocatalytic Experiments

Humic acid solutions were prepared by dissolving humic acid sodium salt (CAS 68131-04-4, Sigma Aldrich, MP > 300 °C) and then adjusting the pH to the desired value using aqueous solutions of NaOH and HCl.

For the determination of the adsorption isotherms, the zirconia powder was added to the HA solutions, and the pH was then adjusted to 6.5. Then, the mixture was kept stirring in the dark for 24 h. The initial humic acid concentration ([*HA*]_0_) was varied between 5 and 20 mg·L^−1^ and the zirconia dosage between 0.1 and 1.0 g·L^−1^.

In the photocatalytic experiments, a specific amount of the zirconia nanoparticles was added to the HA solution to achieve the desired catalyst dosage. As a reactor, a 100 mL borosilicate glass beaker was used. The pH was then adjusted to the desired value. Before irradiation, the mixture was kept under stirring in the dark for 3 h to achieve HA adsorption equilibrium. Then, the mixture was irradiated with visible light for more 3 h under stirring, without any injection of gas. Samples were collected in specific intervals of time. The source of light was a home-built apparatus, consisting of a series of red–green–blue (RGB) light-emitting diodes (LEDs) disposed in a cylindrical support (diameter 11 cm), in which the beaker with the suspension was placed in the center. The LEDs’ emission spectrum is composed of three peaks with maximum intensities around 455, 530, and 625 nm, all in the visible range. The total power of the system was 20 W, and the irradiance at the center of the cylinder was 30 W.m^−2^ (measured with the HD 2302.0 Light meter, Delta OHM). Next, for determining the HA concentration in the solution, the samples were centrifuged at 11,000 rpm for 10 min. The UV–Vis spectra of the supernatants were then recorded using the spectrophotometer UVIKON 930 (Kontron Instruments, Zurich, Switzerland), in the wavelength range of 200 to 700 nm. The concentration of humic acid was then calculated by the UV absorbance at 254 nm. This method has been extensively applied for determining the HA concentration [21,35,36,37,38], and it can be correlated to the total organic carbon (TOC) value through a calibration curve [39,40]. TOC measurements were carried out with the Total Organic Carbon Analyzer TOC-V_CSH_ (Shimadzu, Kyoto, Japan), equipped with an ASI-V auto-sampler, and fed with zero-grade air. The TOC was determined as the difference between total carbon (TC) and inorganic carbon (IC).

The stability of Ce-ZrO_2_-SG nanoparticles was investigated in multiple HA photodegradation cycles. For that, after each 180 min cycle, a concentrated HA solution was added to achieve the initial HA concentration and keep the same initial catalyst dosage (1 g·L^−1^). Then, the dispersion was irradiated again for another 180 min. No treatment, such as washing or centrifugation, was performed between the cycles.

### 2.3. Preparation of the Photocatalitic Membrane and Fouling Tests

For the preparation of the photocatalytic membranes, 100 mg of Ce-ZrO_2_-SG nanoparticles were dispersed in 100 mL of distilled water and ultrasonicated for 10 min. Then, the dispersion was vacuum-filtered to settle the particles in a filter paper support (bleached Kraft paper for sterile packaging applications), with an active filtration area of 12.6 cm^2^. Before the addition of the particles, filter papers were treated with acetone and ammonium hydroxide for cleaning purposes.

**Figure 1:**
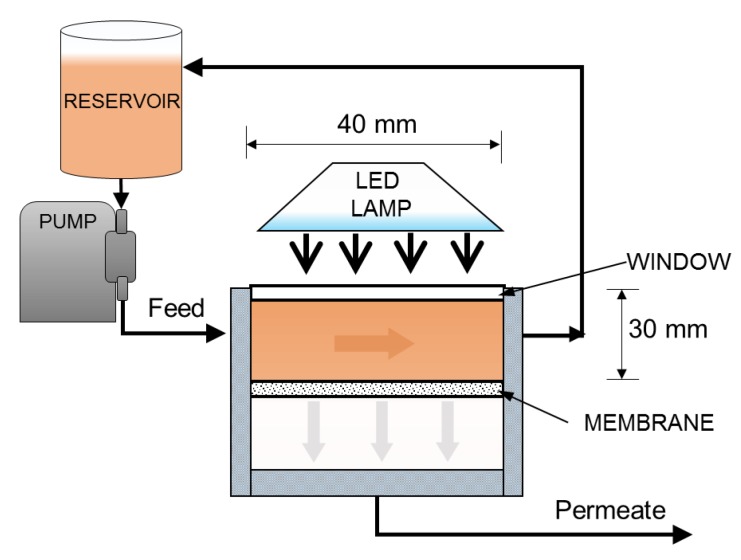
Cross-flow filtration setup for fouling experiments with light irradiation.

Filtration tests were performed in a cross-flow filtration setup made in poly(methyl methacrylate) (PMMA) with a polystyrene window for light irradiation (Figure 1). The height of liquid between the membrane and the window was 3 cm. An aqueous solution of humic acid (15 mg·L^−1^, pH 7) was used as a feed stream. The feed reservoir was connected to the diaphragm pump STEPDOS 08 S (KNF Flodos, Sursee, Switzerland) to keep a cross flow rate of 3.4 L·h^−1^. The mass of permeate obtained was continuously measured with the digital balance KERN 572 (KERN & SOHN Balingen, Germany), and the concentration of humic acid was measured using the UV–Vis spectrophotometer Cary 300 Scan (Varian, Palo Alto, CA, USA). Two sets of fouling experiments were performed using the photocatalytic membranes. In the first set, two separated tests were done, one in the dark and other under irradiation. In the second set, the filtration was performed initially under dark, until the membrane was fouled, and the flux stabilized; then, the filtration was continued under light irradiation. As light source it was used a 18 W white LED lamp, which has a color temperature of 6500 K and no UV/IR emission, as indicated by the manufacturer (WELLMAX, Shanghai, China). The light was positioned just above the window, providing an irradiance of 1000 W·m^−2^.

## 3. Results and Discussion

### 3.1. Characterization of Photocatalysts

In agreement with previously reported characterization procedures [27,33,34,41,42], the structure of the resulting phases, obtained via both syntheses, is a mixture of monoclinic and tetragonal ZrO_2_ (Figure S1). In both pure and doped oxides, the monoclinic phase presents some degree of anisotropy, as suggested by the variable width of the diffraction peaks [34,42]. None of the samples shows the presence of diffraction peaks related to the formation of CeO_2_ phases, indicating that cerium was successfully inserted in the ZrO_2_ matrix.

**Figure 2:**
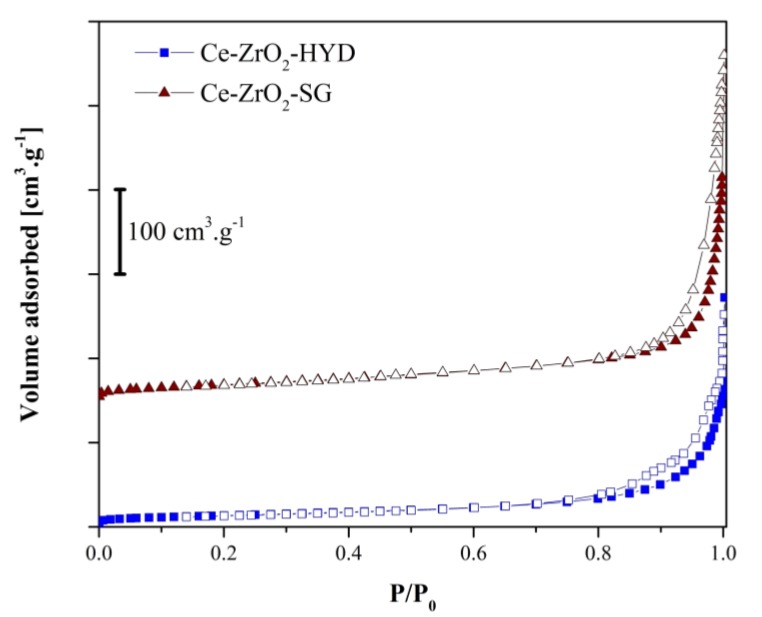
N_2_ adsorption/desorption isotherms of Ce-ZrO_2_ prepared by sol–gel (SG) and hydrothermal (HYD) processes.

To investigate the surface area and porous structure of the doped zirconia samples, nitrogen adsorption/desorption analyses were performed. The BET method was used to determine the specific surface areas of the samples (reported in Table 1). As shown in Figure 2, both samples show type IV IUPAC (International Union of Pure and Applied Chemistry) isotherms with a hysteresis loop type *H*3 (typically observed in aggregates of plate-like particles giving rise to slit-shaped pores) in the relative pressure (*P*/*P*_0_) range of 0.8 to 1.0, which indicates the presence of large mesoporosity. However, it is quite probable that this porosity is due to void space given by interparticle porosity, given by aggregation of primary particles [43,44] rather than intraparticle porosity. The explanation relies on the fact that to accommodate such large pores, bigger particles are required, which are not present in the samples, as suggested by the crystallite size (10–20 nm of diameter) and TEM images obtained in a previous work [33]. Regarding the specific surface area, the doped zirconia prepared by sol–gel synthesis exhibits a higher *S_BET_* than the hydrothermally synthesized sample (Table 1), whereas, the addition of Ce does not cause a substantial modification in the morphology of hydrothermal (HYD) sample but causes a decrease of the specific surface area of the SG sample from 150 to 70 m^2^·g^−1^.

**Figure 3:**
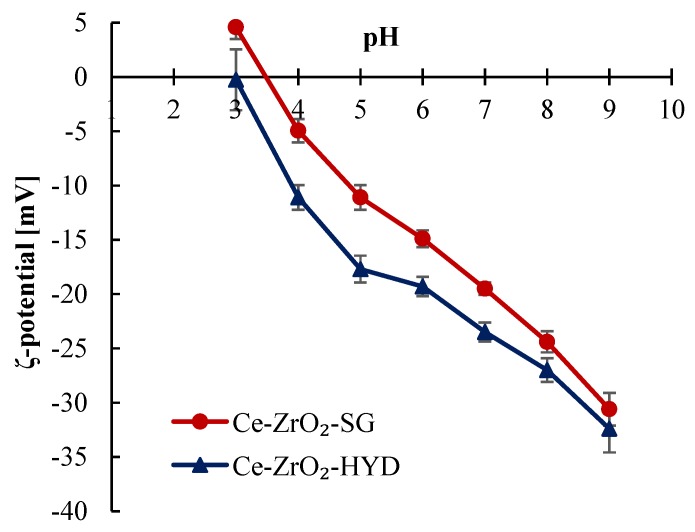
Zeta potential as a function of pH for the Ce-ZrO_2_ prepared by hydrothermal (HYD) and sol–gel (SG) processes.

To evaluate the surface charge of the Ce-doped zirconia samples in aqueous solutions, since it is closely related to the adsorption properties, the zeta potential of the samples was measured at several pH values, as shown in Figure 3. It can be observed that both hydrothermally and sol–gel prepared samples exhibit a negative charge in a wide range of pH (4–9), which indicates that the surface of the zirconia, as expected, is rich in OH groups (vide FTIR spectra, Figure S2). The zirconia OH groups can be classified as terminal or bridged; terminal OH is a Brønsted base site, while bridged OH is a Brønsted acid site resulting from the protonation of bridging oxygen ions [45]. Using titration methods, Sahu and Rao [46] observed the prevalence of acid sites on the zirconia surface, which explains the highly negative zeta potential values measured in the present work when increasing the pH. These authors also stated that the distribution of active sites is closely related to the number of defects, which is mainly affected by preparation methods (e.g., calcination temperature, wet or dry synthesis). This fact can help to understand why the sol–gel prepared Ce-ZrO_2_ has less negative zeta potential values than the hydrothermal one. In fact, the last step in the sol–gel process is calcination at 500 °C, which reduces the number of OH groups in the particles surface.

The interest in these materials in the present paper deals with their optical absorption properties, which were investigated by diffuse reflectance spectroscopy (DRS). As observed in Figure S3, the addition of a small amount of cerium dramatically affects the optical properties of the materials, promoting a red-shift in the absorption spectra. An absorption shoulder, centered at ca. 300 nm, with a tail in the visible region is clearly observed in both hydrothermal and sol–gel Ce-doped samples, but it is more pronounced in the hydrothermal sample, as also observed previously [34]. In regards to the band gap transitions, two *E_gap_* values were reported, 5.2 and 3.6–3.7 [33,47], the first value associated with the fundamental VB→CB transition of ZrO_2_ (unaffected by the Ce doping), the second one due to the absorption band associated to the O 2p→Ce 4f charge transfer transition [41]. Therefore, this result indicates that the prepared Ce-doped oxides have the potential to work as photocatalysts under solar light irradiation.

### 3.2. Adsorption Isotherms

Humic acid is a large molecule (30–50 kDa) with several hydrophilic functional groups, such as COOH and OH, but also with large hydrophobic moieties, some of them consisting of aromatic rings [10]. Given the chemical structure, it can interact very easily and strongly with ceramic materials which are rich in OH groups via ionic, electrostatic, and hydrogen bonding. For our purposes, i.e., the study of antifouling materials for ceramic membrane production, humic substances are interesting for at least two reasons, as already discussed in the Introduction section: on the one hand, several substances present in natural water have structure and behaviors similar to those of humic substances and the study of the fate of these compounds in filtration units can allow to predict how long a membrane can resist the fouling process before the regeneration; on the other hand, humic substances can mimic the fouling substances found in filters after separation of organic molecule (i.e., pollutants) from aqueous matrices. As the regeneration of a membrane requires time and economic burdens, it would be highly convenient to produce a membrane with antifouling properties, which can be done using a material with no affinity towards fouling substances (hydrophobic), or producing a membrane where the fouling can be removed by irradiation in the presence of a proper photocatalyst. If the photocatalytic material is used to fabricate the membrane, one can imagine producing an antifouling membrane with potentially infinite working time and no necessity of cyclic regeneration. Achieving the objective, the functional membrane prepared will be neither subjected to lack of permeation during use and have several advantages for the industrial application of the device.

The photocatalyst we would like to use is the Ce-doped zirconia, whose activation upon visible light irradiation can allow to obtain an integrated system for filtration of pollutants and consequent abatement of the retentate in solar plants.

Since the Ce-doped zirconia nanoparticles are rich in –OH groups (vide FTIR spectra, Figure S2), a considerable adsorption of HA into the particles surface is expected, but it is known in the literature that often the adsorption is only the first step to obtain the photocatalytic abatement of the adsorbed substrates [22,44]. To evaluate the extent of HA adsorption at the surface of Ce-ZrO_2_ nanoparticles, a series of experiments were performed to obtain the related adsorption isotherms (in the dark, pH 6.5, T = 25 ± 3 °C). Then, the Langmuir model, which assumes monolayer adsorption onto a surface containing a finite number of adsorbing sites, and the Freundlich model, which assumes heterogeneous surface energies, were evaluated to fit the experimental data (Figure S4). Only the Langmuir isotherm presented a good correlation (R^2^ > 0.99) with the experimental data. Therefore, the relevant data of Langmuir fit are reported in Table 2. This can be explained by the fact that HA has a highly negative charge at neutral pH [48]; then, HA is mainly adsorbed in a monolayer rather than in multilayers due to the large electrostatic repulsion between the adsorbed molecules and the molecules to be adsorbed [49]. The Langmuir equation is expressed as:

(1) $$\frac{C_{e}}{q}=\frac{1}{q_{e}K_{L}}+\frac{C_{e}}{q_{e}}$$

In this equation, *C_e_* is soluble material concentration [mg·L^−1^], *q_e_* is the adsorption capacity [mg·g^−1^], q is the adsorption rate [mg·g^−1^], and *K_L_* is Langmuir equation constant.

**Figure 4:**
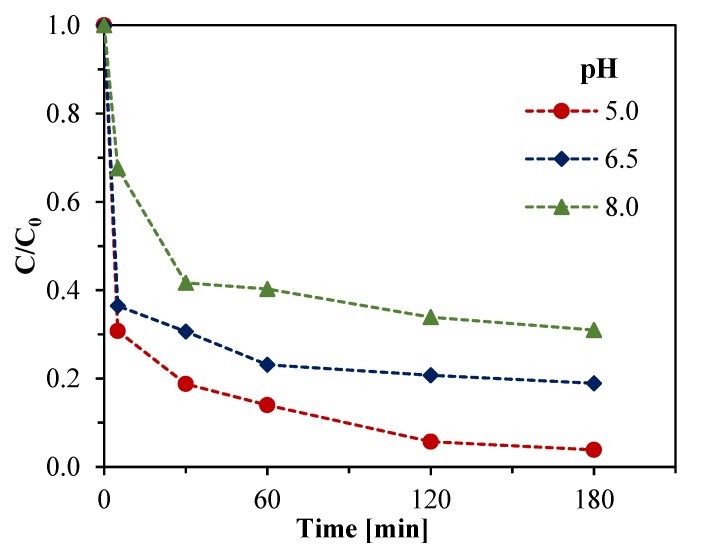
Adsorption of humic acid on Ce-ZrO_2_-SG (under dark, T = 25 ± 3 °C, [HA]_0_ = 10 mg·L^−1^, adsorbent dosage = 1 g·L^−1^).

From Table 2, it can be observed that the sol–gel zirconia has a higher HA adsorption capacity than the hydrothermal one. One possible explanation for this fact is the higher specific surface area of the sol–gel zirconia (Table 1), which implies a higher number of active sites available for the HA adsorption. In addition, the sol–gel prepared ZrO_2_ has a lower negative surface charge than the hydrothermal one (*ζ* = *−17.2 at pH 6.5* vs. *−21.4 mV*); therefore, the repulsion between the negatively charged humic acid and the surface is less intense, favoring the adsorption of HA. To corroborate this statement, it can be shown how the pH affects HA adsorption on Ce-ZrO_2_-SG particles (Figure 4). At pH 5.0, the negative surface charge of zirconia is lower (Figure 3), which reduces the electrical repulsion by the negatively charged HA molecules, favoring their adsorption. However, it should be noticed that, at pH values lower than 5.5, the solubility of HA decreases considerably [50], which also explains the increased HA apparently adsorbed in more acidic solutions. Moreover, in acid medias, humic acid molecules may aggregate to the extent of forming micelle-like structures [51]. On the other hand, at higher pH values, humic acid carboxylic and phenolic group deprotonate, increasing the HA negative charge [48,51]. Consequentially, the HA repulsion exerted by zirconia nanoparticles is greater.

The values obtained for the maximum adsorption using doped zirconia are in agreement with other adsorbents. A value of 8 mg·g^−1^ was reported by Ferro-Garcia et al. [52] for a commercial activated carbon. For zeolites, maximum HA adsorption capacity values of 8.7 and 13 mg·g^−1^ were obtained by Moussavi et al. [53] and Coppola et al. [54], respectively. In comparison with other photocatalysts, Liu et al. [49] obtained an adsorption capacity of 5.9 mg·g^−1^ using zeolite/TiO_2_ particles, whereas, Sun and Lee [48] reported 8 mg·g^−1^ for TiO_2_ microspheres.

### 3.3. Photocatalytic Activity

**Figure 5:**
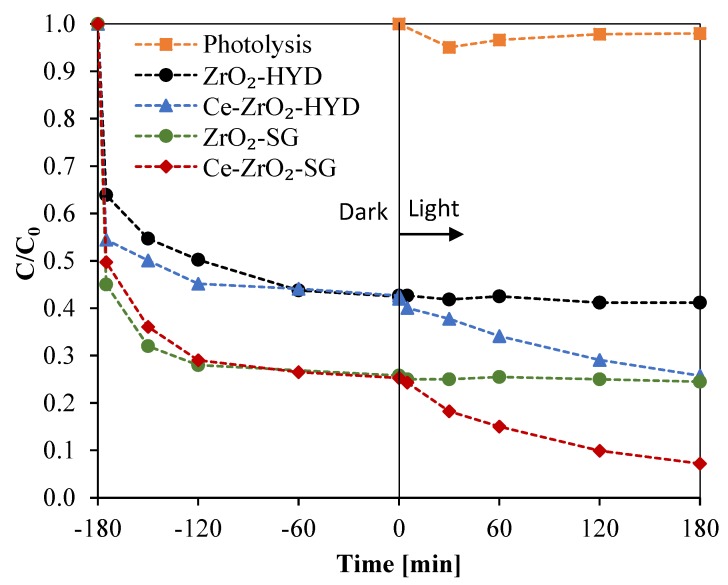
Adsorption and photodegradation of humic acid using different zirconia samples ([HA]_0_ = 10 mg·L^−1^, pH 6.5, catalyst dosage = 1 g·L^−1^).

To evaluate if the prepared zirconia nanoparticles would be able to degrade humic acid in a photocatalytic process, a series of experiments was done with 1 g·L^−1^ of pure and doped ZrO_2._ An experiment without any powder was also performed to check the direct photolysis of HA. Results are shown in Figure 5.

From this figure, it can be observed that practically no degradation of the humic acid occurs under visible-light irradiation, in agreement with results reported by other authors [35,55]. It can also be noticed that in the presence of pure ZrO_2_, the removal of HA was caused only by adsorption. In fact, after reaching adsorption equilibrium, any further removal of HA under irradiation was not observed. This result corroborates the ones obtained by DRS and previous electron paramagnetic resonance (EPR) characterization [27,33,34,42] since pure zirconia presents a wide band gap value of 5.2 eV, and visible-light irradiation is not able to promote the charge separation. Thus, without photo-induced electrons and hole, neither the direct oxidation of HA nor the generation of radicals capable of degrading HA are possible. On the other hand, both hydrothermal and sol–gel Ce-doped ZrO_2_ adsorb HA under dark and are also able to degrade humic acid under visible light irradiation in a photocatalytic process. As discussed in the previous section, the cerium doping introduces in the oxide an intra band gap state based on the Ce 4f levels. Therefore, using a double jump mechanism, Ce-ZrO_2_ can absorb visible light frequencies, promoting charge separation. Then, the photo-induced electrons and holes initiate the HA oxidation reactions. For the Ce-ZrO_2_-SG sample, 93% of the humic acid was removed after 180 min of adsorption in the dark followed by 180 min of light irradiation.

#### 3.3.1. Effect of the Initial HA Concentration

**Figure 6:**
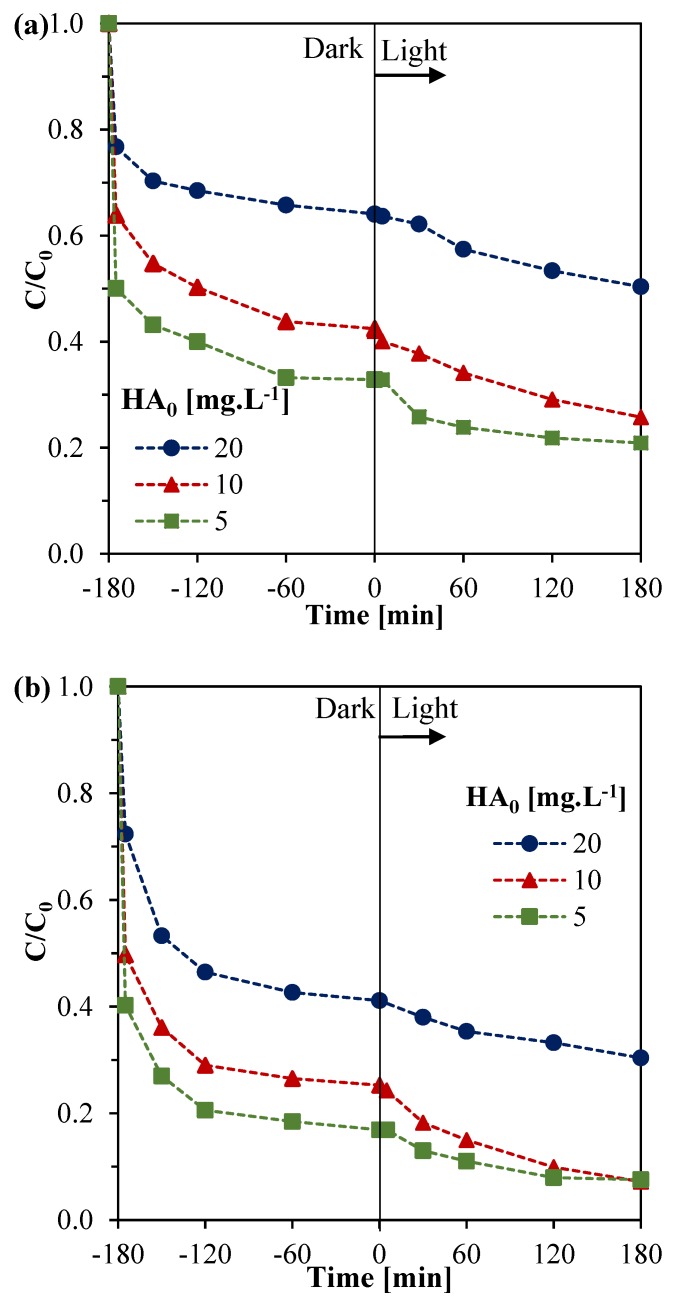
Effect of initial humic acid concentration on humic acid (HA) removal using (**a**) Ce-ZrO_2_-HYD and (**b**) Ce-ZrO_2_-SG (pH 6.5, catalyst dosage = 1 g·L^−1^).

Considering the potential application of the material, it is important to study the dependence of removal efficiency with the initial humic acid concentration ([*HA*]_0_). Depending on the source of water (surface, ground), season, proximity to urban centers, the concentration of organic matter can vary considerably, from a few mg·L^−1^ to more than a hundred. Therefore, the values of initial HA concentration studied were those typically reported in works devoted to advanced oxidation process [21,22,36]. Figure 6 shows the photocatalytic experiment results starting from different initial HA concentrations using 1 mg·L^−1^ of the hydrothermally and sol–gel prepared Ce-ZrO_2_. It can be observed that HA removal decreases increasing the initial HA concentration, as also observed by other authors [35,36]. The explanation relies on the fact that at higher concentrations, more HA molecules are adsorbed at the zirconia nanoparticles surface, reducing the availability of the sites active for the photocatalytic reaction [36] and also increasing the repulsion for non-adsorbed HA molecules [56]. In addition, the adsorbed colored HA molecules may also hinder the absorption of light by the zirconia particles, slowing down the degradation rate. It is also possible that since the same dosage of catalyst was used and consequently the number of radicals generated was the same, a less concentrated HA solution can be degraded more efficiently in shorter time.

The kinetic modelling helps to draw a conclusion about reaction rates and provides useful rate equations for engineering the development of the system. For photocatalytic reactions, especially in the ones where adsorption represents a critical step in the photo-oxidation processes, the Langmuir–Hinshelwood model is the most used [22,45,57,58,59]. This model assumes that both oxidant and reducer are rapidly adsorbed and the rate-determining step of the reaction involves these both species present in a monolayer at the solid–liquid interface [60]. The initial reaction rate, *r*_0_ [mg·L^−1^·min^−1^], in the Langmuir–Hinshelwood model can be expressed as follows:

(2) $$r_{0}=\frac{-dC}{dt}=k\frac{K_{ads}C}{1+K_{ads}C}=k_{obs}C$$

where *k* is the apparent reaction rate constant [mg·L^−1^·min^−1^], and *K_ads_* is the adsorption equilibrium constant [L·mg^−1^]. These constants can be grouped in a lumped parameter to obtain a pseudo-first-order kinetic equation, in which *k_obs_* [min^−1^] is the pseudo-first-order rate constant. Upon integration and linearization of Equation (2), it is possible to determine *k_obs_* by fitting the experimental data (Figure S5). The values obtained are shown in Table 3. With these values, it is possible to calculate the Langmuir–Hinshelwood constants (Table 3) by another linear fit (Figure S6) using the linearized form of Equation (2), as follows:

(3) $$\frac{1}{k_{obs}}=\frac{1}{k}C_{0}+\frac{1}{kK_{ads}}$$

Wang et al. [35] also applied the Langmuir–Hinshelwood modeling to the photodegradation of humic acid. The composite ZnO-TiO_2_-Bamboo charcoal was used as a catalyst. These authors reported a value of 0.22 L·mg^−1^ for *K_ads_*, which is in agreement with the value obtained in the present work, and 0.87 mg·L^−1^·min^−1^ for the apparent reaction rate, *k.* Although this value is one order of magnitude higher than that obtained in our experiments, the measured irradiance applied by those authors was 1500 against 30 w·m^−2^ in the present work, justifying the results.

Comparing hydrothermal and sol–gel prepared Ce-ZrO_2_, the sol–gel sample exhibits higher rate constants (Table 3) and higher removal of humic acid combining adsorption and photodegradation (Figure 6). In addition, several membrane coating techniques are derived from sol–gel chemistry [61,62,63,64]; therefore, considering further application of doped zirconia as an anti-fouling layer in filtration membranes, the rest of the study will be focused on the sol–gel Ce-ZrO_2_.

#### 3.3.2. Effect of the Initial pH

**Figure 7:**
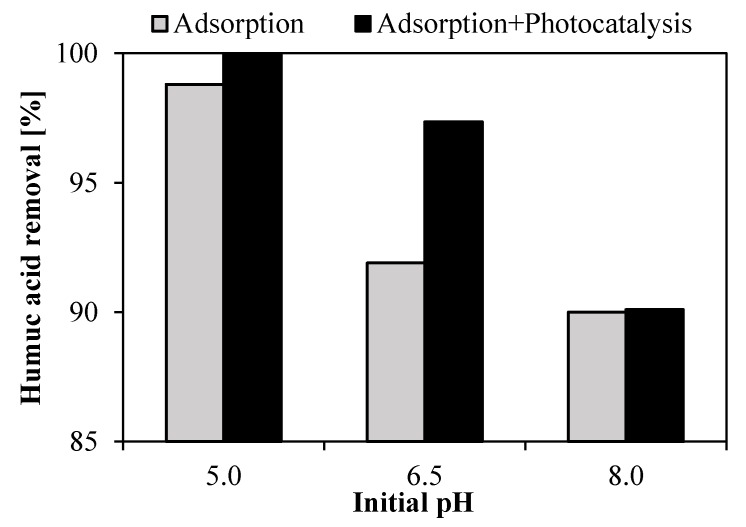
Effect of initial pH on humic acid removal using 1 g·L^−1^ of Ce-ZrO_2_-SG catalyst ([HA]_0_ = 10 mg·L^−1^).

Solution pH is a relevant operational parameter since it governs the protonation/deprotonation of organic compounds and the surface functional groups of zirconia, thus, affecting the efficiency of adsorption and photocatalysis [65]. In addition, elevated costs can arise from pH adjustment, which can determine the feasibility of the processes. Therefore, to investigate the effect of the pH on the photocatalytic degradation efficiency of HA, the values of 5.0, 6.5, and 8.0 wer chosed to work at, which are not so far from neutrality and within the range of natural surface waters [66]. In the experiments, 1 g·L^−1^ of Ce-ZrO_2_-SG was used and an initial HA concentration of 10 mg·L^−1^; the results, as obtained with preliminary adsorption carried out in the dark (adsorption contribution) and with subsequent irradiation (adsorption + photocatalytic removal), are shown in Figure 7.

An increasing trend in HA removal as the acidity increases can be observed, in agreement with other works on photocatalytic degradation of HA [35,36,58]. However, it should be noted that HA adsorption at the catalyst surface, which is an important step in the photodegradation process [58], was also higher at lower pH values. As discussed before, the HA adsorption is intensely affected by the solution pH, since in the acid pH range, the solubility of HA decreases considerably while its negative charge decreases. At the same time, the surface charge of zirconia becomes less negative, favoring the HA adsorption. Regarding the photocatalytic activity, for TiO_2_ based photocatalysts, it has been reported that, under acidic conditions, the catalyst surface is more conducive for electrons leading to production of a photocurrent [67], which contributes to the generation of reactive oxygen species, and meanwhile avoids the recombination of electrons and holes [58]. Therefore, since adsorption is the dominant mechanism in the HA removal [21], the larger difference between adsorption and adsorption + photocatalysis was obtained at pH 6.5, which was used in further experiments.

#### 3.3.3. Effect of Catalyst Dosage

**Figure 8:**
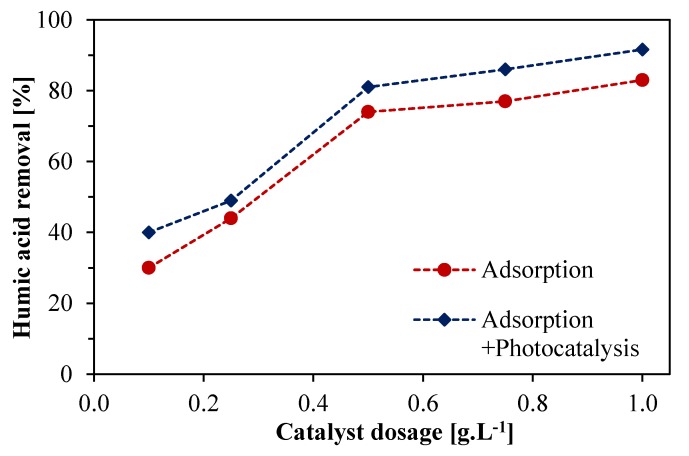
Effect of Ce-ZrO_2_-SG catalyst dosage on humic acid removal (pH 6.5, [HA]_0_ = 10 mg·L^−1^).

The influence of the amount of Ce-ZrO_2_-SG nanopowder on the photodegradation efficiency is shown in Figure 8. The increase of the catalyst dosage leads to an increase of HA removal by both adsorption and photocatalytic mechanisms. Higher doses of catalyst imply higher surface area and number of active sites available, which favors light interaction and HA adsorption [68]. On the other side, it has been reported in some studies, that higher doses of catalyst could lead to negative effects due to light scattering and agglomeration of nanoparticles [36,68,69,70,71,72]. However, at least apparently, these phenomena were not observed in the catalyst dosage range tested, as the trend of adsorption contribution and adsorption + photocatalytic removal remains the same.

### 3.4. Stability of Ce-ZrO_2_ Photocatalyst

**Figure 9:**
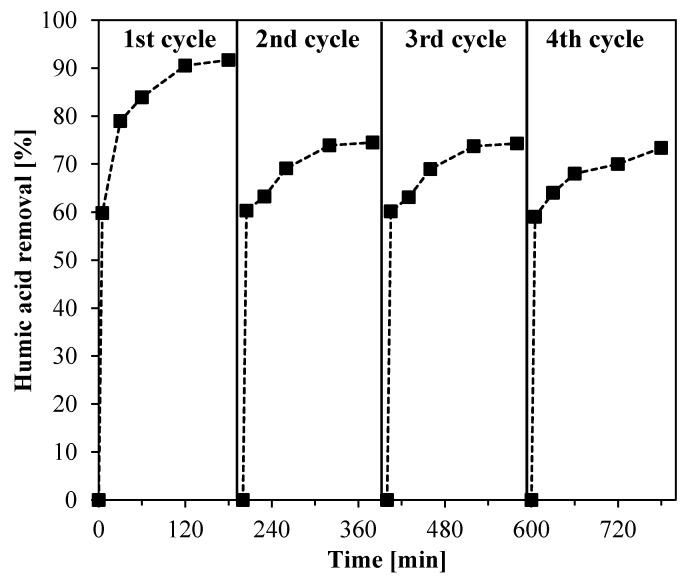
Removal of humic acid in multiple photodegradation tests (Ce-ZrO_2_-SG dosage = 1 g·L^−1^, pH = 6.5, [HA]_0_ = 10 mg·L^−1^).

The stability of photocatalyst and its auto-regeneration capacity is critical for its application in controlling fouling in membrane filtration processes. To demonstrate the potential applicability of Ce-ZrO_2_ photocatalysts, the stability of Ce-ZrO_2_-SG nanoparticles was investigated. Figure 9 shows the results of HA removal in multiple degradation cycles. It can be observed that there is a loss of performance from the first to the second cycle, but the performance keeps relatively stable from the second to the following two photodegradation cycles. It can be presumed that some HA molecules of high molecular weight and aromaticity are not completely degraded, accumulating in the solution. In addition, unreacted HA molecules, adsorbed on zirconia nanoparticles, can block ZrO_2_ active sites and the light absorption. It is worth noting that no treatment, such as washing, was performed between the cycles. At the end of a cycle, a concentrated HA solution was added to achieve the initial HA concentration, and the dispersion was irradiated again for 180 min. Therefore, these results suggest that Ce-doped zirconia have relatively stable performance as the active sites are regenerated under irradiation.

### 3.5. Spectral and TOC Changes in Treated HA Solutions

**Figure 10:**
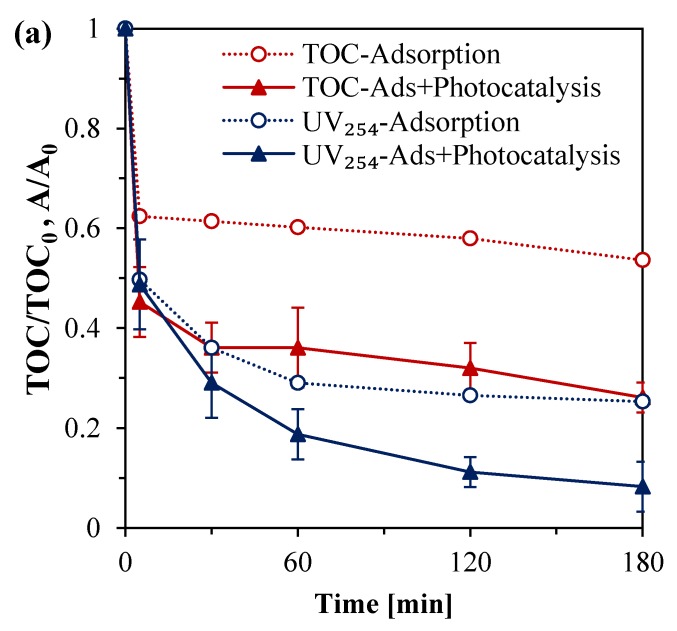

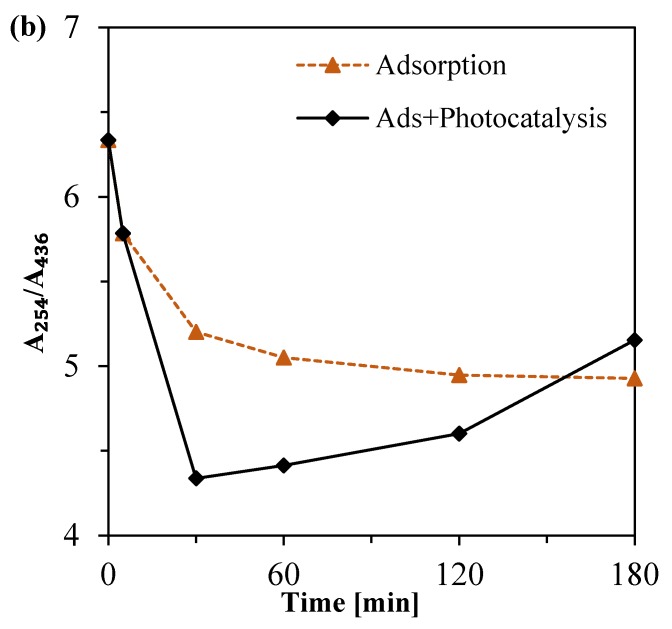
(**a**) Removal of humic acid in terms of total organic carbon (TOC) and UV 254 nm absorbance; (**b**) ratio between UV–Vis absorbance values at the wavelength of 254 and 436 nm (A_254_/A_436_) in HA photodegradation tests (Ce-ZrO_2_-SG dosage = 1 g·L^−1^, pH = 6.5, [HA]_0_ = 10 mg·L^−1^).

In Figure 10a, the removal of humic acid, using Ce-ZrO_2_ in the dark (adsorption) and under visible light irradiation (Ads+Photocatalysts), is presented in terms of TOC and UV absorption at 254 nm (UV_254_). It is worth noting that both UV_254_ absorbance and TOC values are used to quantify the humic acid, but a mathematical correlation of these two values is required to compare their absolute values directly [66]. In addition, the TOC measurements can be considered as preliminary analysis, given the complexity of quantifying the HA by this technique. The determination of a calibration curve and more replicates would be needed to provide more accurate results. Nevertheless, UV–Vis analyses were performed in triplicates and presented with the respective error bars.

From Figure 10a, it can be observed that the TOC reduction is lower than the UV_254_ absorbance reduction, indicating that the HA is probably degraded to intermediates, such as alcohols, aldehydes, ketones, and carboxylic acids [40], higher UV radiation absorbing compounds. Nevertheless, comparing the final TOC values, there is a considerable difference in the values of adsorption and photocatalysis, which is evidence that some degree of mineralization was achieved. These results are in agreement with HA degradation employing as photocatalysts TiO_2_/Fe_2_O_3_/GO [21], TiO_2_/ZnO [35], and ZnO [36].

Uyguner and Bekbolet [40], studying the different molecular weights fraction of humic acid after a TiO_2_ photocatalytic treatment, verified that the ratio between the absorption in wavelengths 254 and 436 nm (A_254_/A_436_) could be used as an indicative parameter of photocatalytic degradation: an increase of the ratio was associated with a reduction in the higher molecular weight fractions of humic acid, which have higher aromaticity than the lighter fraction. Therefore, this ratio A_254_/A_436_ was calculated for the HA photocatalytic degradation with Ce-ZrO_2_ and presented in Figure 10b.

It can be observed that the ratio A_254_/A_436_ presents a fast decrease at the beginning of the experiment in the dark, and then it stabilizes because adsorption equilibrium was reached. This indicates that the HA molecules of lower molecular weight are preferentially adsorbed at the zirconia surface, since higher molecular weight fractions have higher aromaticity [40] and, thus, a more limited interaction with zirconia. Under irradiation, the ratio A_254_/A_436_ first decreases due to HA adsorption and then it increases continuously, since HA molecules have been degraded to lower molecular weight compounds.

### 3.6. Effect of Scavengers—Photocatalytic Mechanism

**Figure 11:**
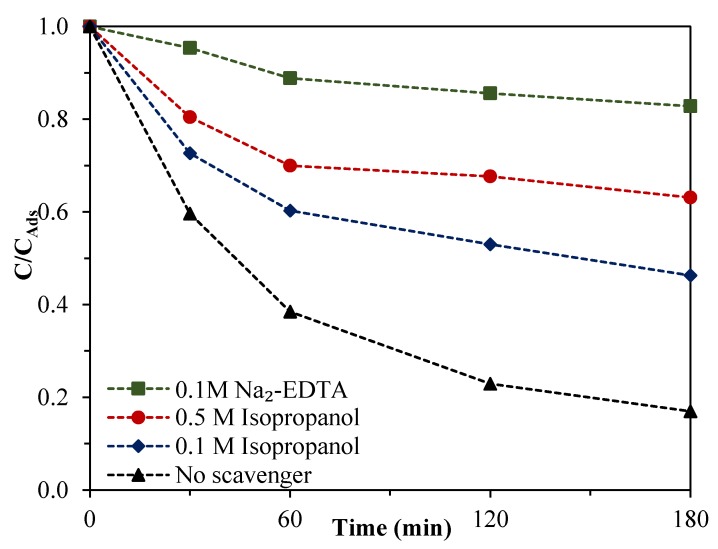
Removal of HA in photocatalytic treatments with and without scavengers. The curves were calculated considering as initial concentration, C_Ads_, the HA concentration after adsorption equilibrium (Ce-ZrO_2_-SG dosage = 1 g·L^−1^, pH = 6.5, [HA]_0_ = 10 mg·L^−1^).

Among the mechanisms proposed for the degradation of organic molecules in photocatalytic processes, two of them have been frequently reported [45,73,74]. In the direct oxidation mechanism, the photo-induced holes oxidize the organic molecule adsorbed on the catalyst. In the indirect mechanism, photo-induced holes and electrons generate reactive oxygen species (ROS), mainly ^•^OH radicals, that degrade the adsorbed organic molecule [45,55,73]. In both cases, adsorption of the organic molecule is critical in the photocatalytic degradation, since photocatalytic reactions proceed at the surface or near the semiconductor surface [45]. In dye bleaching studies, a third mechanism is also reported, in which the dye acts as a photosensitizer by adsorbing the light and generating or initiating the reactions of ROS formation [75]. However, this mechanism does not seem probable for the humic acid photodegradation under visible light, considering that HA solutions do not have higher absorption in the visible region. As discussed in previous works [27,33,34,42], EPR studies indicated the generation of photo-induced holes and electrons, and the formation of ^•^OH radicals when Ce-doped ZrO_2_ was irradiated. Hence, to elucidate HA degradation mechanism with doped zirconia, a series of experiments were performed with disodium ethylenediaminetetraacetate (Na_2_-EDTA) (pointed as scavenger for holes [76] and a chelating agent that prevents HA adsorption on the oxide surface [73,77]) and isopropanol (commonly used as scavenger of ^•^OH radical [77,78]). The results are shown in Figure 11.

In the presence of Na_2_-EDTA, it can be observed that the photocatalytic degradation of humic acid is highly decreased since HA molecules are unable to adsorb at the zirconia surface to undergo the oxidation reactions. Exhibiting a concentration-dependent effect, isopropanol also reduces the HA photodegradation efficacy by quenching ^•^OH radicals. Thus, it can be inferred that ^•^OH radicals are also responsible for the HA oxidation.

**Scheme 1:**
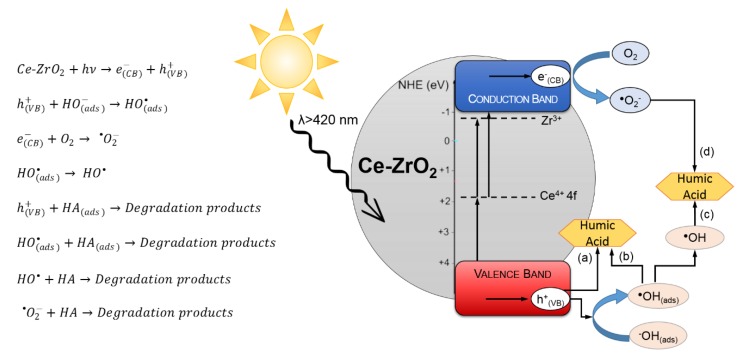
Proposed mechanism for the photodegradation of humic acid (HA) under visible light using Ce-ZrO_2_ as photocatalyst.

Finally, considering previous EPR results [33] and the present observations in the HA degradation a mechanism for the humic acid degradation under visible light irradiation using with Ce-ZrO_2_ as a photocatalyst is proposed in Scheme 1. First, in a double jump mechanism, Ce-doped ZrO_2_ adsorbs visible light photons promoting the photo-induced charge separation. Then, the zirconia valence band trapped holes (h^+^_(VB)_): (a) directly extracting electrons from the adsorbed HA molecules and oxidizing them; and/or (b) reacting with the ^−^OH groups adsorbed on the photocatalyst (^−^OH_(ads)_) producing adsorbed ^•^OH radicals (^•^OH_(ads)_) which successively oxidize HA molecules at the catalyst surface. Finally, in a less likely process, (c) the generated ^•^OH radicals are released from the surface to the solution and oxidize HA molecules distant from the photocatalyst surface. Although this possibility was not evaluated in the tests with scavengers, (d) the superoxide anion radical (^•^O_2_^−^), generated by photo-induced electrons (e^−^_(CB)_), could also degrade the HA.

### 3.7. Filtration Experiments with the Photocatalytic Membranes

**Figure 12:**
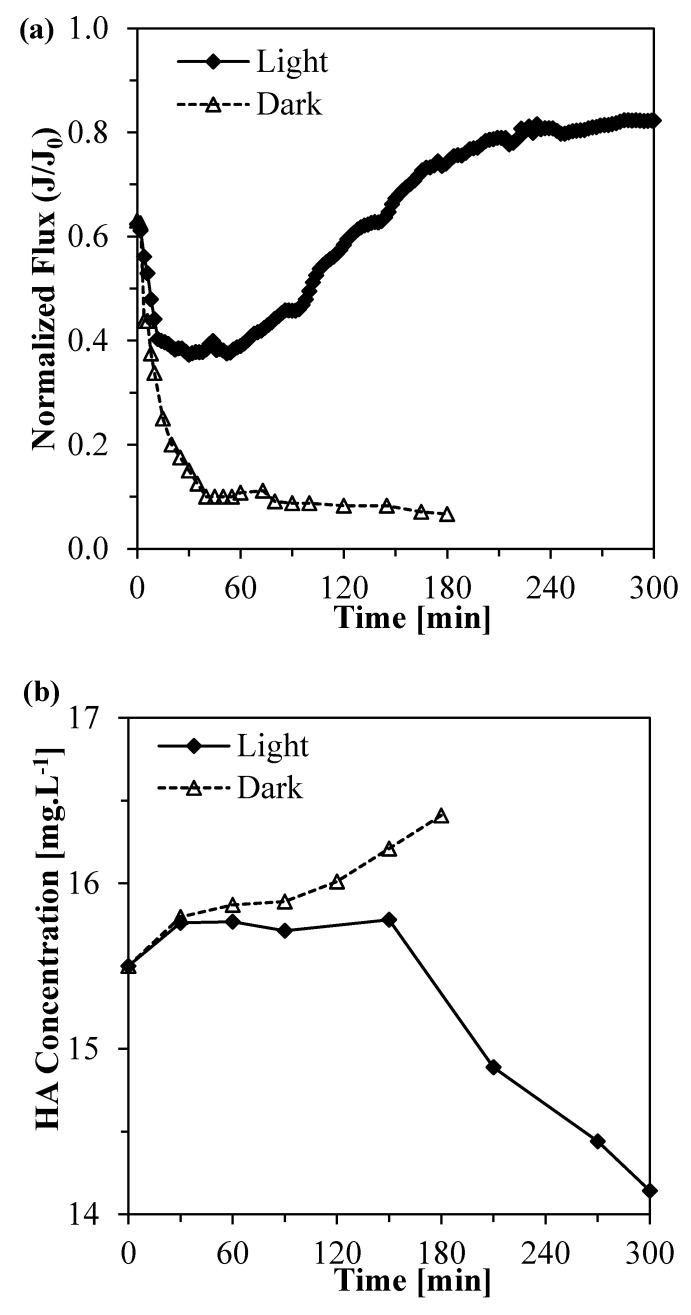
(**a**) Normalized permeate flux and (**b**) HA concentration in the feed stream for HA filtration tests in the dark and under visible light irradiation. (J_0_ = pure water flux = 95.2 L·h^−1^·m^−2^).

**Figure 13:**
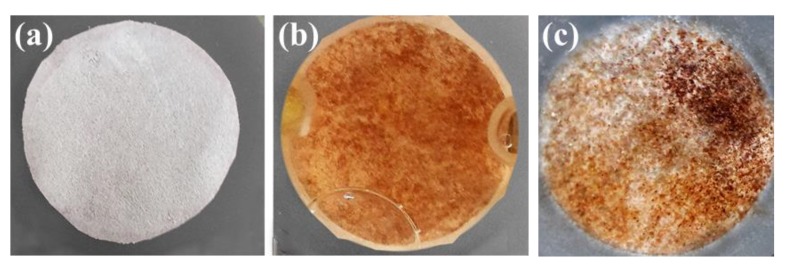
Photocatalytic membranes prepared with Ce-ZrO_2_-SG: (**a**) before filtration, (**b**) after HA filtration in the dark, and (**c**) after filtration under irradiation.

Initially, the flux of pure water in the photocatalytic membrane was measured. A value of 95.2 L·h^−1^·m^−2^ was obtained for the filtration under dark, and this was considered the initial flux J_0_ for further experiments. In the filtration of a humic acid solution (15 mg·L^−1^), under dark conditions, the permeate flux decreased almost 90%, from 60 to 6.3 L·h^−1^·m^−2^, in less than one hour (Figure 12a). This rapid and extensive reduction in the flux can be attributed to irreversible fouling caused by the chemical adsorption of humic acid into the membrane surface [6] since humic acid has a strong affinity with the polar groups [10] present in both ZrO_2_ particles and filter paper. The fouling of the membrane was also visually observed by comparing the membrane before and after the HA filtration, as shown in Figure 13. This figure also shows the membrane after filtration under irradiation, in which a change in the color was observed, but it remained colored since the feed solution still had 14 mg·L^−1^ of humic acid.

The HA retention was around 60% for the experiments in the dark and under irradiation. Therefore, as the feed was recirculated, the concentration of HA in the feed tends to increase during the filtration, as observed in the dark filtration test (Figure 12b).

In the filtration experiment with visible light irradiation (Figure 12a), the flux initially decreased due to the HA adsorption in the membrane; however, after one hour, the flux started to increase, recovering the initial value after another hour. Then, the further increase in the flux may be explained by the degradation of the humic acid in the feed solution. Although it was not evaluated, there is the possibility that under irradiation, the hydrophilicity and wettability of the zirconia surface increases favoring the water flux, which has been reported for TiO_2_ under UV–light irradiation [1].

From Figure 12b, it can be observed that, in the experiment with light, the HA concentration did not increase as in the dark experiment, but it even decreased. This indicates that the Ce-ZrO_2_ nanoparticles were able not only to reduce the fouling but also to photodegrade the humic acid.

**Figure 14:**
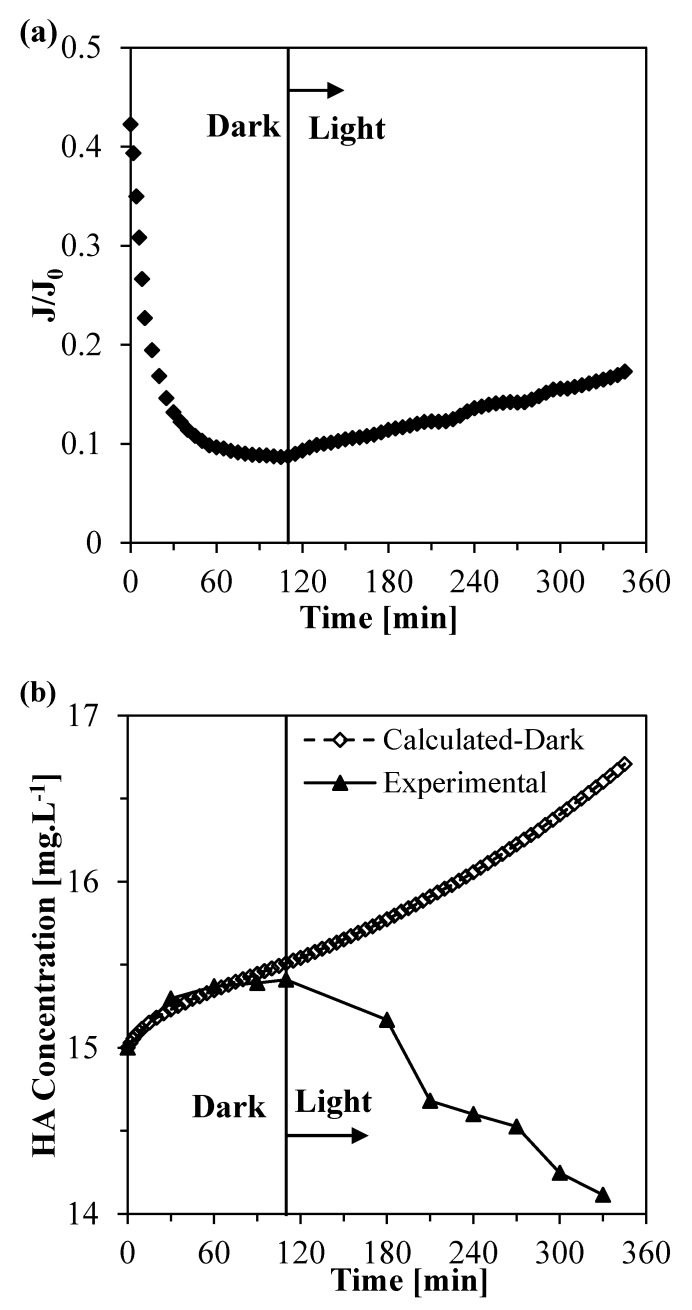
(**a**) Normalized permeate flux and (**b**) HA concentration in the feed stream for HA filtration test initially in the dark and after starting visible light irradiation. (J_0_ = pure water flux = 95.2 L·h^−1^·m^−2^).

To evaluate the possibility of using the light to clean the fouled photocatalytic membranes, initially the filtration was performed in the dark; and then, irradiation was started. In the dark, the permeate flux reduced 80%, as shown in Figure 14a, due to the HA fouling. Measuring the permeate volume and its HA concentration it was possible, through a mass balance, to estimate the HA concentration in the feed under dark conditions (dashed line in Figure 14b), which exhibits an increasing trend. The experimental HA concentration values matched this increasing trend but, after starting light irradiation, the HA concentration declined and the permeate flux started to increase (Figure 14). After 4 h of irradiation, the flux was the double the value after fouling. Therefore, it can be said that the light was able to degrade part of the HA that was fouled in the photocatalytic membrane, promoting the recovery of the flux, and to degrade the HA in the feed stream.

## 4. Conclusions

Ce-doped ZrO_2_ nanoparticles with photocatalytic activity under visible light were synthesized in this study by hydrothermal and sol–gel processes. The addition of a small amount of cerium introduces intra band gap states that act as a bridge between the VB and the CB of zirconia, allowing the absorption of low-energy photons in a double jump mechanism [33,34]. Therefore, a red shift in the absorption spectra is observed, indicating the possibility to use visible light to promote charge separation and the generation of ROS to degrade organic compounds. Thus, this catalyst can be considered a third-generation photocatalyst [79]. Ce-ZrO_2_ was then applied in the photodegradation of humic acid, a good model molecule for membrane fouling tests. As a result, it was observed that the photocatalytic process led to the degradation of HA into smaller molecules, as indicated by spectroscopic and TOC changes. Sol–gel prepared Ce-ZrO_2_ exhibited higher HA removal, achieving 93% of efficiency in 180 min of irradiation using RGB LEDs as light source, after 180 min of adsorption in the dark. Further experiments also confirmed the catalyst activity in neutral pH conditions and its stability for several photocatalytic cycles. Finally, zirconia nanoparticles were applied in the fabrication of photocatalytic membranes, in which it was possible to improve anti-fouling resistance by degrading HA in filtration tests under visible light irradiation. In addition, it was possible to clean a fouled membrane promoting the recovery of the flux. The present findings point to the further development of Ce-ZrO_2_ photocatalytic membranes with improved anti-fouling properties. This will be important for new industrial applications of membrane filtration processes, especially for water treatment. In such processes, solar light photocatalysis can be used to degrade organic fouling compounds and possibly contaminants of emerging concern, which would have remarkable environmental implications. Therefore, this system requires more studies for its optimization and testing with real water samples.

## Figures and Tables

**Table 1:** Specific surface areas (S_BET_) of pure and Ce-doped zirconia samples.

Sample	S_BET_ [m^2^·g^−1^]
ZrO_2_-HYD	44 ± 4
Ce-ZrO_2_-HYD	49 ± 5
ZrO_2_-SG	150 ± 15
Ce-ZrO_2_-SG	70 ± 7

**Table 2:** Parameters for the humic acid (HA) adsorption isotherms in the doped zirconia samples.

Sample	Langmuir Adsorption Model		
	q_e_ [mg·g^−1^]	k_L_	R^2^
Ce-ZrO_2_-HYD	9.3	0.36	0.998
Ce-ZrO_2_-SG	12.3	0.74	0.997

**Table 3:** Pseudo-first-order kinetic rate constants (*K_obs_*) in photocatalytic experiments with different initial HA concentrations ([*HA*]_0_) and the Langmuir–Hinshelwood equation fitted parameters (*k*, *K_ads_*).

Catalyst	[HA]_0_ [mg·L^−1^]	k_obs_ [min^−1^]	r^2^	k [mg·L^−1^·min^−1^]	K_ads_ [L·mg^−1^]
Ce-ZrO_2_-HYD	20	0.0023	0.96	0.058	0.24
	10	0.0049	0.97		
	5	0.0053	0.94		
Ce-ZrO_2_-SG	20	0.0040	0.91	0.099	0.28
	10	0.0097	0.96		
	5	0.0091	0.96		

## Supplementary Materials

The following are available online at https://www.mdpi.com/2079-4991/9/4/534/s1, Figure S1: XRD patterns of pure and Ce-doped ZrO_2_ prepared by hydrothermal and sol-gel processes. Triangles and squares on top of the patterns indicate the peaks of monoclinic (m-ZrO_2_, ICSD #658755) and tetragonal (t-ZrO_2_, ICSD #66781) phases, respectively, Figure S2: FTIR spectra of Ce-ZrO_2_ samples prepared by hydrothermal (HYD) and sol-gel (SG) processes. FTIR spectra were registered in transmission mode using a Bruker Vector 22 spectrophotometer equipped with Globar source, DTGS detector, and working with 128 scans at 4 cm^−1^ resolution in the 4000–400 cm^−1^ range. Samples were analyzed as self-supporting pellets by dispersing the samples in KBr (1:20 weight ratio), Figure S3: (**a**) UV-Vis-DRS spectra of pure and Ce-doped ZrO_2_ prepared by hydrothermal and sol-gel processes, (**b**) magnification of region 200–500 nm, Figure S4: Linear fitting of Langmuir isotherm equation to experimental data of HA absorption on Ce-ZrO_2_ samples prepared by hydrothermal (HYD) and sol-gel (SG) processes, Figure S5: Determination of the pseudo-fist-order kinetic rate constants, K_obs_, for (**a**) Ce-ZrO_2_-HYD and (**b**) Ce-ZrO_2_-SG, Figure S6: Determination of the Langmuir-Hinshelwood model parameters for Ce-ZrO_2_ samples prepared by hydrothermal (HYD) and sol-gel (SG) processes.
