# Supplementary material for: Control of Membrane Fouling in Organics Filtration Using Ce-Doped Zirconia and Visible Light
Source: Nanomaterials (Basel). 2019 Apr 3;9(4):534. doi: 10.3390/nano9040534 (PMC6523972; doi:10.3390/nano9040534)
Supplement: Supplementary file 1 [file nanomaterials-09-00534-s001.pdf]

nanomaterials

Supplementary Material

Control of Membrane Fouling in Organics Filtration Using Ce-Doped Zirconia and Visible Light

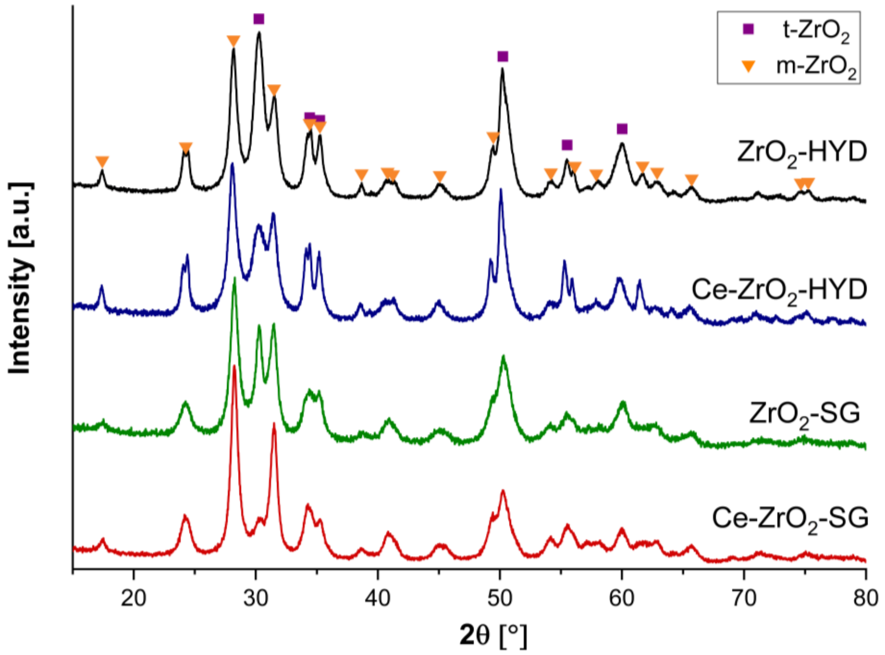

Figure S1. XRD patterns of pure and Ce-doped ZrO_2 prepared by hydrothermal and sol-gel processes. Triangles and squares on top of the patterns indicate the peaks of monoclinic (m-ZrO_2, ICSD #658755) and tetragonal (t-ZrO_2, ICSD #66781) phases, respectively.

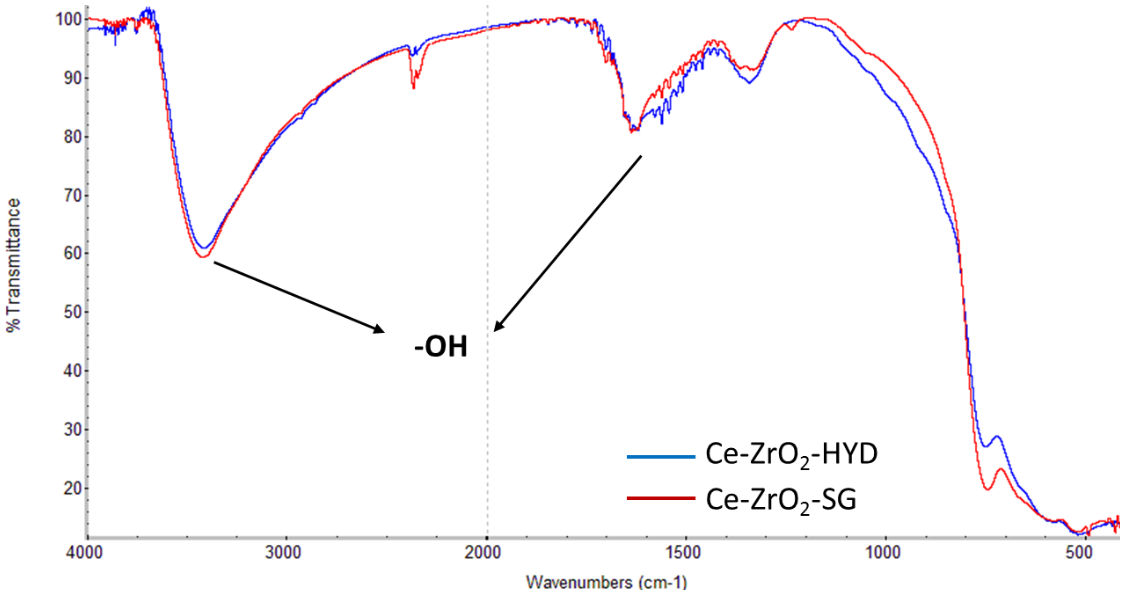

Figure S2. FTIR spectra of Ce-ZrO2 samples prepared by hydrothermal (HYD) and sol-gel (SG) processes. FTIR spectra were registered in transmission mode using a Bruker Vector 22 spectrophotometer equipped with Globar source, DTGS detector, and working with 128 scans at 4 cm^{-1} resolution in the 4000–400 cm^{-1} range. Samples were analyzed as self-supporting pellets by dispersing the samples in KBr (1:20 weight ratio).

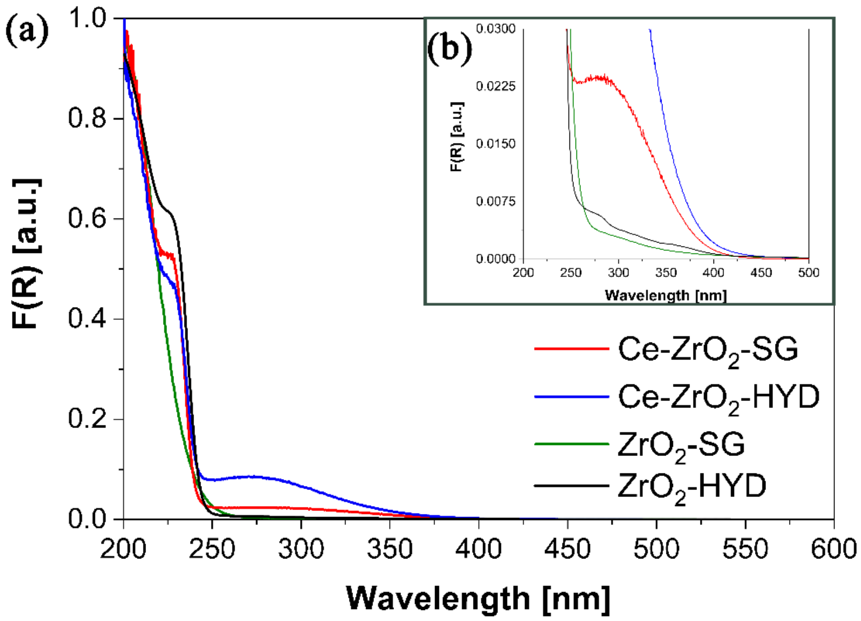

Figure S3. (**a**) UV-Vis-DRS spectra of pure and Ce-doped ZrO_2 prepared by hydrothermal and sol-gel processes, (**b**) magnification of region 200–500 nm.

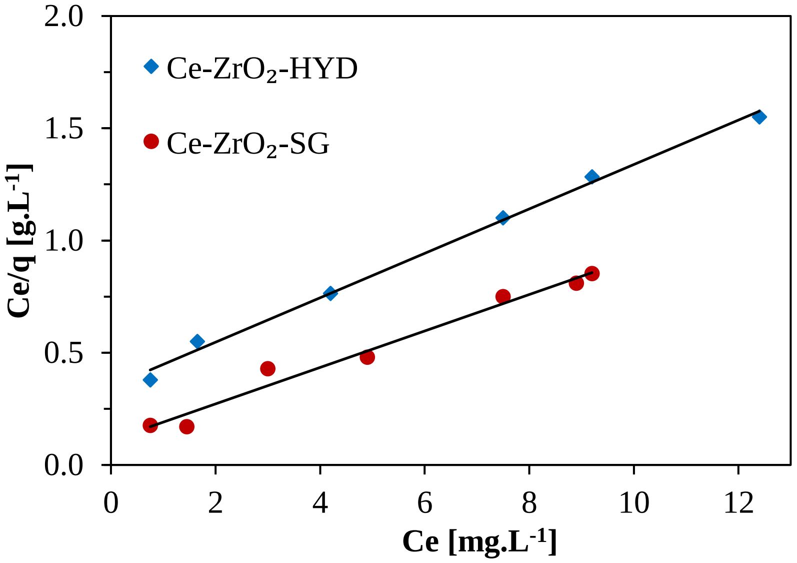

Figure S4. Linear fitting of Langmuir isotherm equation to experimental data of HA absorption on Ce-ZrO_2 samples prepared by hydrothermal (HYD) and sol-gel (SG) processes.

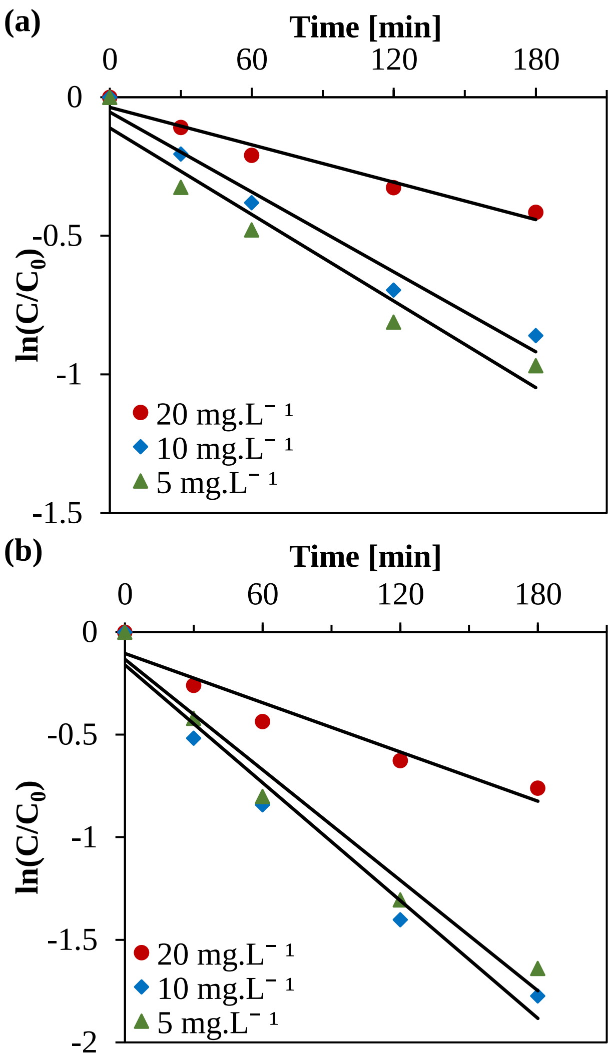

Figure S5. Determination of the pseudo-fist-order kinetic rate constants, K_{obs}, for (**a**) Ce-ZrO_2-HYD and (**b**) Ce-ZrO_2-SG.

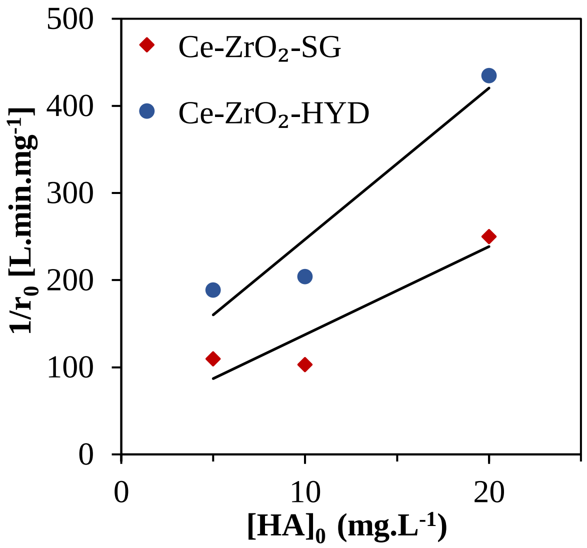

Figure S6. Determination of the Langmuir-Hinshelwood model parameters for Ce-ZrO_2 samples prepared by hydrothermal (HYD) and sol-gel (SG) processes.
